# Multimodal E-Mental Health Treatment for Depression: A Feasibility Trial

**DOI:** 10.2196/jmir.1370

**Published:** 2010-12-19

**Authors:** David C Mohr, Jennifer Duffecy, Ling Jin, Evette J Ludman, Adam Lewis, Mark Begale, Martin McCarthy Jr

**Affiliations:** ^2^Group Health Research InstituteGroup Health CooperativeSeattleUnited States; ^1^Department of Preventive MedicineNorthwestern UniversityChicagoUnited States

**Keywords:** Depression, Internet, feasibility, telephone, telemedicine

## Abstract

**Background:**

Internet interventions for depression have shown less than optimal adherence. This study describes the feasibility trial of a multimodal e-mental health intervention designed to enhance adherence and outcomes for depression. The intervention required frequent brief log-ins for self-monitoring and feedback as well as email and brief telephone support guided by a theory-driven manualized protocol.

**Objective:**

The objective of this feasibility trial was to examine if our Internet intervention plus manualized telephone support program would result in increased adherence rates and improvement in depression outcomes.

**Methods:**

This was a single arm feasibility trial of a 7-week intervention.

**Results:**

Of the 21 patients enrolled, 2 (9.5%) dropped out of treatment. Patients logged in 23.2 ± 12.2 times over the 7 weeks. Significant reductions in depression were found on all measures, including the Patient Health Questionnaire depression scale (PHQ-8) (Cohen’s *d* = 1.96, *P* < .001), the Hamilton Rating Scale for Depression (*d* = 1.34, *P* < .001), and diagnosis of major depressive episode (*P* < .001).

**Conclusions:**

The attrition rate was far lower than seen either in Internet studies or trials of face-to-face interventions, and depression outcomes were substantial. These findings support the feasibility of providing a multimodal e-mental health treatment to patients with depression. Although it is premature to make any firm conclusions based on these data, they do support the initiation of a randomized controlled trial examining the independent and joint effects of Internet and telephone administered treatments for depression.

## Introduction

Epidemiological studies have shown that 6.7% to 10.1% of the general population suffers from a depressive or mood disorder in a 12-month period [1,2]. Over the past 8 years, a number of Internet interventions have been developed to treat depression [3]; however, these interventions have often resulted in high rates of attrition [4]. (Note that in this paper, attrition and adherence refer to dropping out or staying in treatment and not to loss to follow-up for study assessments.) For example, studies examining open access to stand-alone Internet treatments find that fewer than 10% of patients return after a first visit and as few as 1% complete treatment [4]. Studies that use recruitment and screening procedures consistent with face-to-face trials find better adherence for stand-alone sites, although the mean number of log-ins remains in the 2 to 4 range for a 7- to 8-module intervention [5,11]. Many studies have found that email support can significantly improve adherence [3]. For example, a trial comparing two different Internet treatments supported by coaching emails reported that between 56% and 72% of participants completed 4 sessions, although only approximately 38% completed the entire Internet treatment [6]. Adherence and outcomes for Internet interventions are generally poorer for depression compared with Internet interventions for other mental health problems such as anxiety disorders [3,4]. This is perhaps not surprising, given that depression reduces motivation for and compliance with recommendations for traditional treatments as well [7,8]. Thus, while adherence to Internet treatments is a problem generally [9], it appears to be more common among patients with depressive disorders.

This paper describes the results of a feasibility trial of a multimodal e-mental health intervention, in which an Internet treatment for depression was supported by brief weekly calls from a “coach.” These calls were designed to maximize adherence to the Internet intervention for depression.

We used 3 basic frameworks in designing the e-mental health and telephone intervention. First, we designed the structure of the website based on the persuasive technologies framework [10], which outlines the principles through which computer technology can be used to persuade individuals to change their behavior. The following principles were used: (1) Simple, brief tools that provide monitoring and feedback are most likely to be useful. Indeed, length and complexity of Internet modules have been suggested as one cause of dropout [11]. (2) Technologies that can be inserted into daily routine are more likely to be persuasive and to promote adherence. Accordingly, most of the patient’s work on the website was designed to require less than 3 to 5 minutes and involved self-monitoring and feedback. The site and the coach suggested website visits daily or every other day. (3) Tools should simplify tasks, in this case those associated with behavioral activation and cognitive restructuring. (4) Feedback should be tailored to the individual. (5) Media such as video can be effective at promoting vicarious learning. (6) Users view interactive websites as social actors. Thus, specific features that affect human relationships can also improve acceptability of a website. These include attractiveness of the site, “personality” of the site, (eg, whether information is provided in a highly directive or more nondirective manner), credibility, and reciprocity (eg, whether the site provides something of value in return for asking the user to provide information or engage in tasks).

Second, Internet interventions for depression show better outcomes and adherence when accompanied by human support. While the role of human support in promoting adherence has been repeatedly confirmed [3,4], there is surprisingly little theoretical or empirical exploration in psychology or psychiatry into why or how human support improves adherence. We have begun developing a framework for understanding how human support increases adherence. This is based on a branch of accountability theory, which describes specific mechanisms by which adherence can be obtained [12]. Accountability refers to the implicit or explicit expectation that an individual may be called upon to justify his or her actions or inactions. Borrowing from accountability theory, the following basic principles would likely be important in a protocol for maintaining adherence via human support. Providers must: (1) be seen as trustworthy, benevolent, and having the necessary expertise, (2) frame the relationship as one containing reciprocity in which the patient can expect to receive definable benefits from the coach, (3) be specific about which outcomes are expected, (4) focus expectations on processes rather than outcomes, (5) monitor adherence and inquire when the patient is nonadherent, (6) specify accountability processes at the beginning of treatment with adequate justification and with patient agreement, and (7) reward and encourage success in meeting goals. We therefore developed an interface to allow a provider or coach to monitor patient activity on the site. We also developed a coaching protocol based upon these principles.

Third, different telecommunications technologies vary in their potential to sustain adherence and outreach [13]. Internet interventions on their own are associated with high attrition [3], likely because these interventions rely principally on patient initiative for engagements and have limited capacity for outreach. The addition of email to these interventions improves adherence and outcomes [3,4]. Telephone interventions are associated with adherence rates of more than 92% [14]. Accordingly, we delivered the coaching protocol via telephone and email.

This paper reports on the adherence and depression outcomes from the feasibility testing of a multimodal eHealth intervention for depression. Our goal was to examine feasibility in terms of recruitment, adherence, and depression outcomes. Secondary outcomes included anxiety and positive affect. We also examined the relationship between adherence and depression outcomes.

## Methods

### Participants

From December 11, 2008, through March 25, 2009, participants were recruited through advertisements posted on a popular online community, Craigslist.org, which features classified advertising. Recruitment was conducted in Chicago, Illinois, USA. Those who were interested in participating were directed to the study Web page where they completed the Patient Health Questionnaire depression scale (PHQ-8) [15]. Respondents who scored 10 or above on the PHQ-8 were invited for a telephone screening interview. Those who were interested in participating received a “verbal informed consent,” in which a research assistant described the study over the telephone, as well as a written consent, which was emailed and could be electronically signed. This study was approved by the Northwestern University Institutional Review Board.

The inclusion criteria were: (1) a score of 10 and above on the PHQ-8, as this is the criterion recommended by the MacArthur Depression Group for referral to psychotherapy or counseling [16], (2) possession of an email account, (3) access to a telephone, (4) access to a computer with broadband access to the Internet, (5) ability to speak and read English, and (6) age 18 years or older. Participants were excluded if they (1) had a hearing or voice impairment that would prevent participation in the coaching sessions and study assessments, (2) had visual impairments that would prevent the use of the website and completion of study assessment materials, (3) met screening criteria for dementia, (4) had a severe psychiatric disorder, including psychotic disorders, bipolar disorder, bulimia or anorexia, or posttraumatic stress disorder (PTSD), (5) reported severe suicidality (eg, plan and intent) or had a history of suicide attempt in the past five years, (6) were currently or planning to receive psychotherapy during the 7-week treatment, (7) had initiated treatment with an antidepressant in the last four weeks, or (8) planned to be out of town for 2 weeks or more during the 7-week treatment phase. 

### Study Design

This was a single arm feasibility trial. Because this is the first test of a novel intervention, a single arm trial testing feasibility rather than efficacy is an appropriate design [17]. Eligible patients were provided log-in information for the moodManager website and were contacted by their assigned coach. Outcomes were assessed at baseline, mid-treatment, and end of treatment (week 7).

### Intervention

#### The moodManager Website

The depression management skills training website, which we called “moodManager,” was based on cognitive behavioral principles [18,19] and consisted of six learning modules and four tools. The learning modules were intended to teach basic concepts of cognitive behavioral therapy and to participants how to use the tools. Content included text, video, and audio material. Modules were designed to require 15 to 20 minutes to complete. Learning modules (and associated tools) included the following: (1) “Getting Started,” which was an introduction to the basic principles of cognitive behavioral therapy (CBT); (2) “Monitoring Activities,” which described the relationship between activities and mood and introduced the “Activity Diary” tool, which allowed participants to track and rate daily activities; (3) “Scheduling Positive Activities,” which taught participants to use the “Activity Scheduler,” a tool used to plan and schedule positive activities; (4) “Identifying Thoughts,” which described the effects of thoughts on mood and taught participants to use the “Thought Diary” tool to monitor automatic thoughts; (5) “Challenging Thoughts,” which expanded the Thought Diary tool by teaching participants to develop alternative thoughts; (6) “Maintaining Gains,” which summarized the skills learned and encouraged participants to continue using the tools for relapse prevention. Tools were designed to support implementation of cognitive behavioral skills, to require only a few minutes to complete, and were intended to be completed every day or every other day. Tools were partially “scaffolded”: the Activity Monitoring tool was incorporated into the Activity Scheduling tool, and the Thought Diary tool was incorporated into the Challenging Thoughts Diary tool. Participants were required to complete a tool three times before the next learning module would open, to ensure that the concept was learned. The moodManager site included mood rating and tracking features for self-monitoring by patients.

The moodManager site also contained a provider interface that allowed the coach to observe the patient’s activity on the site, including dates and times of site visits, content of patient’s entries into tools (eg, activity diaries and thought records), depression monitoring ratings, and alerts for suicidality.

#### The Coaching Protocol

Participants were assigned a coach who contacted them once per week by telephone and once per week by email. Based on principles of accountability theory [12], self-determination theory [20,21], and motivational interviewing, we designed a brief telephone coaching program that we called Telephone Coaching to Support Adherence to Internet Interventions (TeleCoach). In accordance with the theory and principles described in the ”Introduction” section above, TeleCoach included the following elements. Coaches must (1) present themselves as trustworthy, benevolent, and having the necessary expertise in coaching an online intervention, (2) frame the relationship as one containing reciprocity in which the patient can expect to receive definable benefits from the coach, (3) be specific about which outcomes are expected (eg, logging into moodManager), (4) monitor adherence through the coach interface and inquire when the patient is nonadherent, (5) specify accountability processes at the beginning of treatment (eg, that the coach can see when the patient uses the site) with adequate justification and with patient agreement, and (6) reward and encourage success in adherence.

TeleCoach included an initial “engagement session,” which was intended to last approximately 30 minutes while subsequent conversations with the coach were intended to be 5 to 10 minutes in length. Participants were also permitted to email their coaches with questions during the week. (A copy of the TeleCoach protocol is available at http://www.preventivemedicine.northwestern.edu/researchprojects/telecoach.htm.)

#### Coaches

The two coaches were PhD-level licensed psychologists. While the protocol is designed so as not to require specialized training, a higher level of expertise was required at this early developmental stage to ensure patient safety. Coaches were blind to outcome assessments but did have access to mood tracking within the moodManager site.

### Measures

#### Adherence

Adherence was measured in two ways. Adherence to the Internet portion of the intervention was defined by the number of log-ins. Adherence to the TeleCoach program was defined by whether or not the coaching phone call was completed. Dropout from the intervention was determined to have occurred at the last point of contact with the website or coach, if it occurred more than one week before the end of treatment period.

Study assessments were completed at baseline, week 4, and week 8. Consistent with standard clinical trial methodology in depression, assessments were performed both by clinical interview administered via telephone and self-report administered online. The telephone interviews were administered by trained clinical evaluators who were blind to the coaching component of the study, site usage, and other data collected by moodManager, such as the depression ratings. Research assistants received extensive training on assessment protocols. All telephone evaluations were audiotaped and reliabilities were periodically checked by having two or more clinical evaluators rate the same tapes (see report of reliabilities below). To ensure separation of the treatment and the evaluation, self-reported outcomes were administered via SurveyMonkey.com separately from the moodManager program. To minimize loss to follow-up, participants were paid up to US $80 for completion of assessments. Participants were clearly informed that payment was not for use of the website to ensure that payments did not influence treatment adherence.

#### Mini International Neuropsychiatric Interview

The Mini International Neuropsychiatric Interview (MINI) [22] is a structured interview to evaluate Diagnostic and Statistical Manual of Mental Disorders, 4th edition (DSM-IV) Axis I disorders, including Major Depressive Episode (MDE), as well as most other major diagnoses. The full MINI was administered by telephone at screening, but only the MDE section was administered at follow-up telephone evaluations.

#### Hamilton Rating Scale for Depression

The Hamilton Rating Scale for Depression (HRSD) was administered at all time points to provide an objective, interview-based measure of depressive symptom severity consistent with trials of face-to-face psychotherapy and pharmacotherapy. A telephone administered version of the 17-item HRSD, developed for the Medical Outcomes Study [23], was administered by a clinical evaluator. Clinical evaluators’ average interrater reliability, using interclass correlations, was .95 (range .81-.99).

#### Personal Health Questionnaire

The Personal Health Questionnaire (PHQ-9) [24] is a 9-item self-report measure of depressive symptoms that closely matches the Diagnostic and Statistical Manual of Mental Disorders, Fourth Edition (DSM-IV) criteria for MDE and was administered online at all time points. To address concerns raised by the IRB, the PHQ-8 [15], which excludes the item measuring suicidality, was used for screening only, prior to patient enrollment in the study. The PHQ-9 was used as the self-report outcome measure.

#### Perceived Barriers to Psychological Treatment

The Perceived Barriers to Psychological Treatment (PBPT), administered at baseline, is a 25-item measure that identifies barriers to access to face-to-face psychological care [7]. It has been shown to predict decreased utilization of face-to-face services.

#### The moodManager Depression Tracking

In contrast to the HRSD and PHQ-9, which were administered independent of the treatment site, this assessment was administered to the patient at each site visit to moodManager. Also, unlike all other assessments, coaches had access to the depression tracking tool ratings and were expected to review them. Patients were asked to rate the two questions (mood and anhedonia) on a 0 to 10 Likert scale, where 0 = no symptoms and 10 = worst possible symptom. These items were adapted from the PHQ-2 [25]. The assessments were intended to help the participant track mood during treatment and were not intended for outcome assessment; however, they were used in secondary analyses as described below. The total depression assessment score had a potential range of 0 to 20.

#### Generalized Anxiety Disorder Scale

The Generalized Anxiety Disorder Scale (GAD-7) [26] is 7-item self-report measure of anxiety symptoms that closely matches the DSM-IV criteria for GAD.

#### Positive Affect

We included only the 10 self-report items from the Positive Affect Scale of the Positive and Negative Affect Scale (PANAS) [27].

#### Telephone Interview for Cognitive Status

The Telephone Interview for Cognitive Status (TICS) is a widely used telephone assessment that has demonstrated reliability and validity in identifying dementia resulting from Alzheimer’s and stroke [28]. It was administered at screening.

#### Demographics

Demographics were collected via online self-report.

#### Exit Interview

Upon completing the final assessments, an unstructured interview was conducted in which patients were asked to review each of the components of the site and intervention, to describe specific problems or critiques, and to provide us with a general overview. These data were collected primarily to assist the team in improving future versions of moodManager and the intervention.

### Statistical Methods

All analyses were conducted on an intent-to-treat basis. Outcome and utilization analyses were conducted by random intercept mixed-effects regression models using the maximum likelihood method for continuous outcome and generalized estimating equations model by SAS Proc Genmod procedure (SAS Institute Inc, Cary, NC, USA) with an exchangeable working correlation structure for binary outcomes. For analyses using moodManager tracking, depression measures were averaged over a one-week period. For the purposes of these analyses, missing data were assumed to be missing completely at random and therefore were ignored.

## Results

### Recruitment

**Figure 1:**
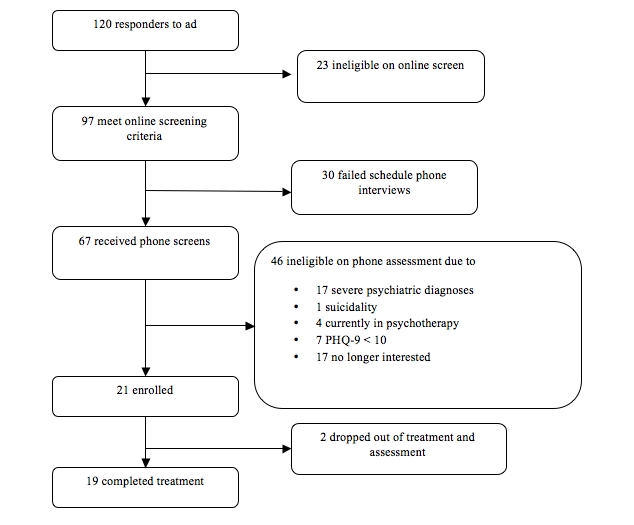
Flow of patients through recruitment, screen, and intervention

During recruitment, 120 individuals responded to the online classified ad, 97 met preliminary criteria and were contacted via email for an eligibility assessment, and 21 were enrolled. The flow of patients through the study is displayed in Figure 1.

### Participant Characteristics

Baseline characteristics of participants can be found in Table 1. Of the 21 enrolled patients, 17 identified barriers on the PBPT that would prevent access to face-to-face treatment.

**Table 1:** Demographics

Demographics		Measure n = 21
Age, mean (SD)		32.90 (9.97)
Female, n (%)		17 (81%)
Married, n (%)		5 (24%)
**Race**		
	African American, n (%)	3 (14%)
	Asian, n (%)	4 (19%)
	Caucasian, n (%)	14 (67%)
**Diagnoses**		
	Major depressive disorder, n (%)	17 (81%)
	Agoraphobia, n (%)	4 (19%)
	Social phobia, n (%)	1 (5%)
**Highest level of education**		
	Completed high school, n (%)	1 (5%)
	Some college, n (%)	4 (19%)
	Associate’s degree, n (%)	1 (5%)
	Bachelor’s degree, n (%)	9 (43%)
	Master’s degree, n (%)	4 (19%)
	Advanced degree, n (%)	2 (10%)
**Current employment status**		
	Employed, n (%)	14 (67%)
	Unemployed, n(%)	4 (19%)
	Disability, n (%)	1 (5%)
	Declined to respond, n (%)	2 (10%)

Among patients who met preliminary screening criteria (n = 97), patients who were enrolled (n = 21) did not differ significantly from patients who were not enrolled on any available data including age (*P* = .43), gender (*P* = .53), race (*P* = .50), or score on PHQ-8 (*P* = .59).

### Outcome Measures

#### Adherence

In total, 19 (91%) participants completed all treatment modules and continued to log in to moodManager throughout all weeks of the treatment. Of 21 participants, 2 (less than 10%) did not complete the intervention; 1 dropped out following the initial engagement session with the coach but without logging on to the website and the other participant dropped out after the 3rd week. Patients who dropped out of treatment also refused to continue assessments and were lost to follow-up.

The mean number of visits to the site over the course of the 7-week treatment program was 23.16 ± 12.16 (Range 7-49). Visits were more frequent in the first week (3.8 ± 1.33) and declined over time but continued through the final (7th) week (2.0 ± 1.28). The 19 patients who completed treatment also completed all 8 TeleCoach calls. In addition, 5 (26%) continued to log in to the site after completion of the trial.

#### Outcomes for Depression, Anxiety, and Positive Affect

The means, standard deviations, and effect sizes for outcome measures are shown in Table 2. Significant improvements were seen in all measures, including the HRSD ( *t*_37_ = -5.37, *P* < .001), the PHQ-9 ( *t*_37_= -7.37, P < .001), the GAD-7 (*t*_37_= -7.48, P < .001), the PANAS-PA (*t*_37_= 5.10, P < .001) and MDE (beta = -2.27, Z = -4.70, P < .001). At baseline, 17 of 21 (81%) patients met criteria for MDE, with 5 of 19 (24%) at week 4, and 1 of 19 (5%) at week 8.

**Table 2:** Means and standard deviations of outcome measures

Measure	Baseline Mean (SD) (n = 21)	Week 4 Mean (SD) (n = 19)	Week 8 Mean (SD) (n = 19)	Effect Size (Cohen’s *d*)	*P*
HRSD	21.2 (6.42)	14.4 (6.41)	12.0 (6.54)	1.34	< .001
PHQ-9	15.0 (3.70)	8.2 (5.18)	5.6 (5.74)	1.96	< .001
GAD-7	10.8 (4.59)	5.3 (3.23)	3.6 (3.85)	1.70	< .001
PANAS-PA	20.6 (5.27)	27.6 (7.74)	29.4 (9.14)	1.16	< .001

### Relationship Between Adherence and Depression

#### Site Visits and Depression.

We examined the week-to-week relationship between utilization and depression by examining the number of site visits and the moodManager depression tracking assessment obtained at each site visit. Poorer mood was associated with a greater number of site visits during the same week (Estimate = 0.11, *t*_95_ = 3.06, *P* = .003). When weeks were lagged so that depression predicted number of site visits in the following week, there was no significant effect (*P* = .91). When depression was lagged to predict subsequent site visits, there was also no significant effect (*P* = .17).

#### Coaching and Depression

The mean amount of time for the initial engagement calls was 28.5 ± 7.22 minutes, and the mean amount of time for subsequent calls was 9.9 ± 6.40 minutes. The length of the coaching call was not significantly related to scores on the moodManager depression tracking tool during the following week (*P* = .36). Similarly, moodManager depression ratings did not predict the length of the following coaching call (*P* = .63).

#### Coaching and Utilization

Length of the coaching call was not significantly related to subsequent number of site visits *(P* = .23), and number of site visits did not significantly predict the length of the coaching call (*P* = .15).

### Exit Interview

Overall, participants reported that the site was helpful and enjoyable to use. Tracking features, specifically activity scheduling and mood ratings, were reported as highlights of the site. Patients also consistently liked the content of the learning modules, and some indicated that more content would be welcomed. General problems reported were mostly usability related issues including critiques regarding navigation and confusion related to rules and requirements for progression through the program. Some patients did not always use tools as taught (eg, many patients used thought records as a free form “journal”), although these participants were still enthusiastic about using the features.

## Discussion

This study examined the feasibility of a multimodal treatment approach to depression. The Internet treatment required more frequent log-ins than are typically required. The telephone coaching support was developed based upon accountability theory and is, to the best of our knowledge, the first manualized telephone support program for an Internet intervention. The trial showed good overall treatment adherence. The attrition rate of under 10% is similar to the 8% rate of attrition seen in telephone administered therapies [14] and compares favorably with the 25% to 50% attrition rates seen in face-to-face psychotherapy and the 35% to 90% attrition rates seen in stand-alone and email supported Internet interventions [4]. In addition, log-in rates to the website averaged nearly 4 per week in the early phase of treatment and remained at twice per week in the final week. While the numbers of log-ins in the later weeks were somewhat lower than recommended, the mean of 23 log-ins over 7 weeks in treatment is, as best we know, higher than other Internet treatments have achieved. Reductions in both diagnosis of depression and severity of depressive symptoms were in the range seen in randomized controlled trials (RCTs) of psychotherapy and antidepressant medications [29,30]. Overall, the finding that patients adhered to the treatment at a high rate and improved suggests that using multiple telecommunications technologies to support Internet interventions for depression is feasible and acceptable to patients.

We examined within treatment relationships between depression and adherence variables. Poorer mood in any given week was associated with a greater number of site visits. However, mood did not significantly predict the number of site visits the following week, nor did number of site visits predict mood. While it is difficult to make inferences about causality, we speculate that poorer mood increased utilization since the alternative, that utilization decreased mood, is inconsistent with the overall positive outcomes. Thus, these findings suggest that patients may titrate their treatment to meet their own needs.

The TeleCoach intervention protocol was designed and developed so that it is easy to learn and ultimately could be used by providers who do not have any specific training in cognitive behavioral therapy, or in psychotherapy more generally. The intent is that nurses, social workers, or other persons serving in the role of care managers could be trained to implement the protocol. The coaching sessions were intended to be 5 to 10 minutes. In fact, the session length mean was approximately 10 minutes. There was considerable variability in length, with many sessions being considerably shorter but many also being much longer. However, there was no support for the ideas that session length was a response to poorer mood or that the length of the session affected mood. Indeed, our experience was that the “chattiness” of the patient was the principal driver of session length.

The patients who enrolled in the study met criteria for anxiety disorders at a much higher rate than seen in the general population [1]. This finding, which was unexpected but not surprising, suggests that Internet interventions may reach populations who commonly are not seen in standard face-to-face psychological interventions. For example, patients with agoraphobia might find it difficult to get to a clinic, while patients with social anxiety may find it uncomfortable to engage in psychotherapy. Indeed, this was borne out by spontaneous comments from patients to the coaches indicating that they would never seek help from a therapist. A posthoc analysis found no evidence that patients with symptoms of agoraphobia or social anxiety performed any better or worse than patients without these symptoms. The potential for Internet interventions to reach such undertreated or untreated populations remains an area with great research and clinical potential.

The primary impetus for this study was the problem of attrition in Internet intervention. Adherence to Internet interventions has repeatedly been identified as a major problem requiring the development of a “science of attrition” [9,31]. Much of the work on attrition has focused on understanding how the reach of the Internet might increase enrollment of patients at greater risk of attrition, incorporating components into trial design to prevent this effect (eg, through the use of run-ins), and understanding patient characteristics associated with attrition [4]. In addition, there have been numerous attempts to reduce attrition through modifications to Internet interventions, but this has been done without much theoretical underpinning. These modifications have included the addition of postcard reminders, email, telephone calls, and online discussion groups [32,33]. While initial attempts to find solutions to the problem of attrition may benefit from investigator intuition and trial-and-error approaches, the area of Internet intervention has advanced to a stage where it could benefit from more refined and better specified models, which define the components of human interaction and support that contribute to adherence. Such a conceptualization could be used to develop more refined intervention solutions to the problem of attrition.

### Limitations

This study is a preliminary feasibility trial and, as such, it has all of the problems associated with such studies. Without a control condition, we cannot rule out the possibility that the improvements in depression were due to the natural course of the illness or to other factors independent of the intervention. Without controlling for telephone and/or website treatments alone, it is also impossible to determine the relative contribution of each to either adherence or depression outcomes. Given the large number of people who did not follow up after initial contact, it is possible that there was some sample selection bias. While the numbers selected out are similar to those seen in other trials of Internet interventions for depression [32,34], the possibility that our results are due to selection bias cannot be ruled out. In addition, the small size of the sample increases the likelihood that the sample is not representative of the population from which we recruited. Finally, while study staff clarified at each assessment point that payment was for assessments and not for moodManager use, it is possible that the adherence to the treatment was influenced by these payments.

### Conclusions

Given these limitations, we emphasize that the results of this feasibility trial should in no way be used to suggest that the moodManager website or the TeleCoach support have any effect on depression or adherence. However, these results are very promising. As the first study of a manualized coach support program for an Internet intervention, this study opens a line of research that has the potential to improve our understanding of how to enhance adherence to Internet interventions. Based on the encouraging results of this study, we have initiated a pilot 3-arm RCT comparing (1) moodManager and TeleCoach, (2) moodManager alone, and (3) a wait-listed control group for the treatment of major depressive episode.

## Abbreviations

CBT: cognitive behavioral therapy
DSM-IV: Diagnostic and Statistical Manual of Mental Disorders, 4th edition
GAD-7: Generalized Anxiety Disorder Scale
MDE: Major Depressive Episode
MINI: Mini International Neuropsychiatric Interview
PANAS: Positive and Negative Affect Scale
PHQ-8: Patient Health Questionnaire depression scale
PTSD: posttraumatic stress disorder
RCT: randomized controlled trial
TICS: Telephone Interview for Cognitive Status
